# Trend and Cancer-Specific Prevalence of Kidney Stones Among US Cancer Survivors, 2007–2020

**DOI:** 10.3390/curroncol32090498

**Published:** 2025-09-05

**Authors:** Chao Cao, Ruixuan Wang, Xiangren Wang, Mohammad Abufaraj, Thomas Waldhoer, Geoffrey T. Gotto, Shahrokh F. Shariat, Lin Yang

**Affiliations:** 1 Department of Medical Oncology, Dana-Farber Cancer Institute, Boston, MA 02215, USA; ruixuan_wang@dfci.harvard.edu; 2 Department of Health Service Research, Management and Policy, College of Public Health and Health Professions, University of Florida, Gainesville, FL 32611, USA; 3 Department of Urology, Medical University of Vienna, 1009 Vienna, Austria; dr.abufaraj@gmail.com (M.A.);; 4 Division of Urology, Department of Special Surgery, Jordan University Hospital, The University of Jordan, Amman 11942, Jordan; 5 Department of Epidemiology, Center for Public Health, Medical University of Vienna, 1009 Vienna, Austria; thomas.waldhoer@meduniwien.ac.at; 6 Southern Alberta Institute of Urology, Department of Surgery, University of Calgary, Calgary, AB T2N 1N4, Canada; 7 Department of Urology, University of Texas Southwestern Medical Center, Dallas, TX 75390, USA; 8 Department of Urology, Weill Cornell Medical College, New York-Presbyterian Hospital, New York, NY 10032, USA; 9 Department of Cancer Epidemiology and Prevention Research, Cancer Care Alberta, Calgary, AB T2N 5G2, Canada; 10 Departments of Oncology and Community Health Sciences, Cumming School of Medicine, University of Calgary, Calgary, AB T2N 1N4, Canada

**Keywords:** cancer survivor, NHANES, kidney stones

## Abstract

**Purpose:** To evaluate the prevalence and cancer-specific patterns of kidney stones among U.S. cancer survivors compared to non-cancer adults. **Methods:** This was a serial cross-sectional, descriptive epidemiologic analysis of a US nationally representative sample from the National Health and Nutrition Examination Survey from 2007 to 2020. Weighted prevalence of kidney stones was estimated for both non-cancer adults and cancer survivors by study cycle. Multivariable logistic regression was conducted to examine factors associated with higher probability of kidney stones in both non-cancer adults and cancer survivors. **Results:** From 2007–2008 to 2017–2020, kidney stone prevalence rose in both non-cancer adults (8.5% to 9.2%, *p* for trend = 0.013) and cancer survivors (13.1% to 17.3%, *p* for trend = 0.033). Throughout the study period, prevalence was consistently higher in cancer survivors. The overall prevalence from 2007 to 2020 was 15.8% (95% CI: 14.0–17.5%) in cancer survivors and 9.2% (95% CI: 8.8–9.6%) in non-cancer adults. After adjusting for sociodemographic, lifestyle, and health factors, cancer survivors had higher odds of kidney stones (OR = 1.28, 95% CI: 1.10–1.49). Compared with non-cancer adults, survivors of ovarian (OR = 3.71, 95% CI: 1.77–7.78), kidney (OR = 2.88, 95% CI: 1.46–5.68), bone and soft tissue (OR = 2.86, 95% CI: 1.12–7.30), uterine (OR = 1.94, 95% CI: 1.17–3.22), cervix (OR = 1.68, 95% CI: 1.08–2.61) and prostate (OR = 1.41, 95% CI: 1.06–1.87) cancers were statistically more likely to report kidney stones. The prevalence was numerically highest among survivors of kidney cancer (34.7%), followed by bone and soft tissue (29.9%), ovarian (29.8%), and testicular (26.3%) cancers. **Conclusions:** The higher prevalence of kidney stones in cancer survivors, with substantial variation by cancer type, highlights the urgent need for effective clinical management of kidney stones in oncology settings and mechanistic research.

## Simple Summary

Kidney stones are a painful and common condition, but their impact on people who have survived cancer is not well understood. This study analyzed national health survey data from 2007 to 2020, including over 40,000 adults without cancer and nearly 3500 cancer survivors in the US. We found that kidney stones became more common over time in both groups, but cancer survivors consistently had higher rates. By 2020, 17% of cancer survivors had experienced kidney stones, compared to 9% of non-cancer adults. Survivors of certain cancers, such as ovarian, kidney, bone and soft tissue, uterine, cervical, and prostate cancers, had especially high rates. Even after accounting for factors like age and health status, cancer survivors were still more likely to report kidney stones. These findings highlight the need for better kidney stone prevention and care strategies for cancer survivors, especially those at highest risk based on their cancer type.

## 1. Introduction

The population of cancer survivors is growing due to early detection and advances in treatment, with a projected 26 million cancer survivors in the United States by 2040 [1]. Approximately 69% of survivors now live five or more years post-diagnosis, and many face persistent health challenges due to the range of acute, long-term, and late effects of cancer and cancer treatment [1,2]. Combined with existing health risk factors, cancer survivors may experience dramatic physiological damage, such as impaired bone metabolism, endocrine disruption, and kidney injuries, which lead to kidney stone formation [3,4,5]. Despite survivors of many cancers having a heightened risk of kidney stones, the prevalence of kidney stones and cancer-specific patterns in the US cancer survivor population remains unknown.

Cancer survivors live with several shared risk factors for kidney stones, such as advanced age, metabolic abnormalities, unhealthy lifestyle behaviors, and chronic conditions [4,6,7]. Previous studies have suggested a higher risk of certain cancers associated with kidney stones, including renal cell carcinoma, renal pelvis/ureter cancer, and bladder cancer [8,9]. Moreover, cancer treatments such as chemotherapy and radiation therapy can damage kidney function, leading to subsequent kidney problems and stone formation [10]. Hence, the burden of kidney stones may vary across different malignancies, owing to their distinct therapeutic modalities and patient characteristics [10]. There is thus a pressing need to understand cancer survivor populations at high risk of kidney stones to inform the development of targeted preventive and management strategies.

To address these knowledge gaps, this descriptive epidemiologic study aimed to examine the prevalence and patterns of kidney stones by sociodemographic and lifestyle factors in non-cancer adults and cancer survivors using data from the National Health and Nutrition Examination Survey (NHANES) from 2007 to 2020.

## 2. Methods

The National Health and Nutrition Examination Survey (NHANES), a major program of the National Center for Health Statistics (NCHS), has surveyed nationally representative samples of the civilian noninstitutionalized US population in 2-yr cycles since 1999 [11]. All the NHANES protocols were approved by the National Center for Health Statistics ethics review board, and written informed consent was obtained from all participants. This modeling investigation was exempt from review because it used published deidentified data sets that included no personally identifiable information. This study followed the Strengthening the Reporting of Observational Studies in Epidemiology (STROBE) Statement guidelines.

### 2.1. Study Population

Participants completed in-person interviews and physical examinations in a mobile examination center. This analysis aggregated data on kidney stones, sociodemographic characteristics, lifestyle factors, and medical conditions among cancer survivors and non-cancer adults aged 20 years or older from NHANES 2007–2020 through 2017–March 2020 before the pandemic.

### 2.2. Diagnosis of Cancer

Data on cancer diagnosis and cancer type were collected during the in-person interview, including cancer type(s) with up to 3 diagnoses and age at each diagnosis. Participants were asked, “Have you ever been told by a doctor or other health professional that you had cancer or a malignancy of any kind?” Individuals who responded yes were defined as cancer survivors and were then asked, “What kind of cancer was it?” and “How old were you when this cancer was first diagnosed?” Years since the first cancer diagnosis was calculated as the difference between the current age and the age at the first diagnosis. Participants with nonmelanoma skin cancer or skin cancer of unknown type were excluded. Cancer type was classified according to the first cancer diagnosis, including cancers of the oral cavity and pharynx, upper gastrointestinal tract, colorectal, larynx/trachea, lung, bone and soft tissue, melanoma, breast, cervix, uterus, ovary, prostate, testis, bladder, kidney, brain, thyroid, lymphoma, blood and leukemia, and other sites [12].

### 2.3. Assessment of Kidney Stones

Information on kidney stones was collected during the in-person interview related to kidney conditions. Participants who responded “yes” to the question, “Have you ever had kidney stones?” were defined as having kidney stones; otherwise, they were classified as not having kidney stones [13].

### 2.4. Sociodemographic Characteristics, Lifestyle Factors, and Chronic Conditions

Self-reported sociodemographic characteristics included age, sex (male vs. female), race and ethnicity (non-Hispanic White, non-Hispanic Black, Hispanic, and non-Hispanic Other [American Indian/Native Alaskan/Pacific Islander, Asian, multiracial]), and educational level (<high school, high school/GED, and >high school). Family income was classified using the ratio of family income to the federal poverty level (<1.3, 1.3–3.5, and >3.5), which follows standard NHANES categorizations based on U.S. federal assistance program eligibility. A higher family poverty income ratio indicated a higher family income status [14]. Participants’ weight and height were measured during physical examinations at the mobile examination center, and body mass index (BMI) was calculated as weight in kilograms divided by height in meters squared. BMI was categorized into three groups: <25, 25.0–<30, and ≥30 kg/m^2^. Lifestyle factors included smoking status (never, former, and current smoker), leisure-time physical activity (inactive, insufficiently active, and sufficiently active), and daily sitting time (<4, 4–<8, and ≥8 h per day). Chronic conditions included diabetes mellitus, cardiovascular diseases (congestive heart failure, coronary heart disease, myocardial infarction, and stroke), and gout, defined by self-reported diagnosis from a health professional. For female participants, self-reported history of pregnancy and hormone therapy use were recorded. Dietary data included total energy intake and daily intake of calcium, sodium, water, and alcohol [13].

### 2.5. Statistical Analyses

All analyses adhered to NHANES analytic guidelines to generate nationally representative estimates. Sample size (weighted%) and prevalence (95% CIs) of kidney stones were calculated for non-cancer adults and cancer survivors overall, by study cycle, and by cancer type among cancer survivors. Linear trends in the prevalence of kidney stones from 2007 to 2020 were evaluated using linear regressions in non-cancer adults and cancer survivors, respectively, across survey cycles. The *p* values for trends were estimated using the survey cycle as a continuous variable. The overall prevalence of kidney stones between 2007 and 2020 was compared by participant characteristics. Weighted logistic regression models were used to estimate odds ratios (ORs) and 95% CIs for correlates of kidney stones in non-cancer adults and cancer survivors, respectively. Forest plots were produced to illustrate multivariable-adjusted odds ratios and 95% CIs for kidney stones, comparing cancer survivors with noncancer adults by participants’ characteristics and cancer types. All statistical analyses were performed using Stata version 17.0 (StataCorp LLC, College Station, TX, USA), applying the “svy” commands to account for the complex survey design and sampling weights of NHANES. Statistical tests were two-sided, with significance defined as a *p*-value less than 0.05.

## 3. Results

A total of 40,395 non-cancer adults (weighted population: 210,662,296) and 3492 cancer survivors (weighted population: 17,607,702) were included in the present analyses (Table 1). Cancer survivors were more likely to be over 65 years old, female, non-Hispanic White, have higher levels of education and family income, higher BMI, a history of smoking, physical inactivity, and a history of diabetes mellitus, cardiovascular diseases, and gout compared to non-cancer adults.

### 3.1. Trend and Prevalence of Kidney Stones in Cancer Survivors and Non-Cancer Adults

**Figure 1:**
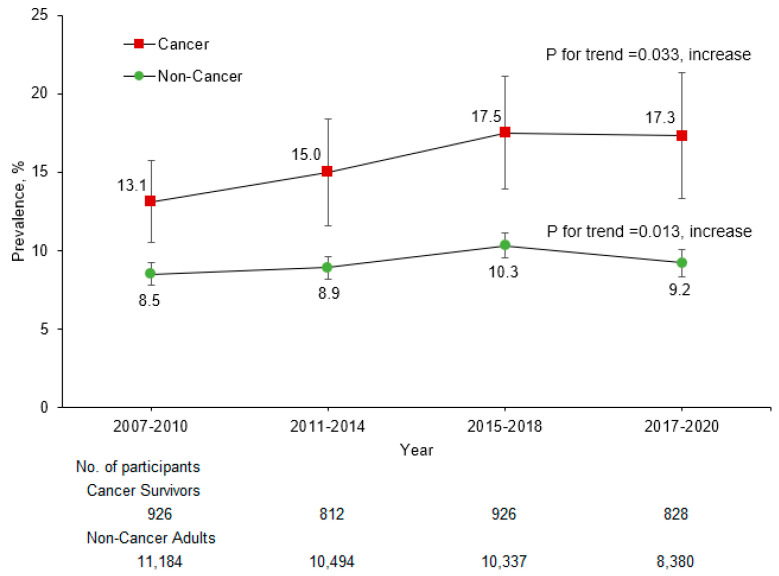
Trends in Kidney Stones Among US Non-Cancer Adults and Cancer Survivors, NHANES 2007–2020 ^a^. ^a^ Estimates were weighted to be nationally representative. Error bars indicate 95% CIs.

**Figure 2:**
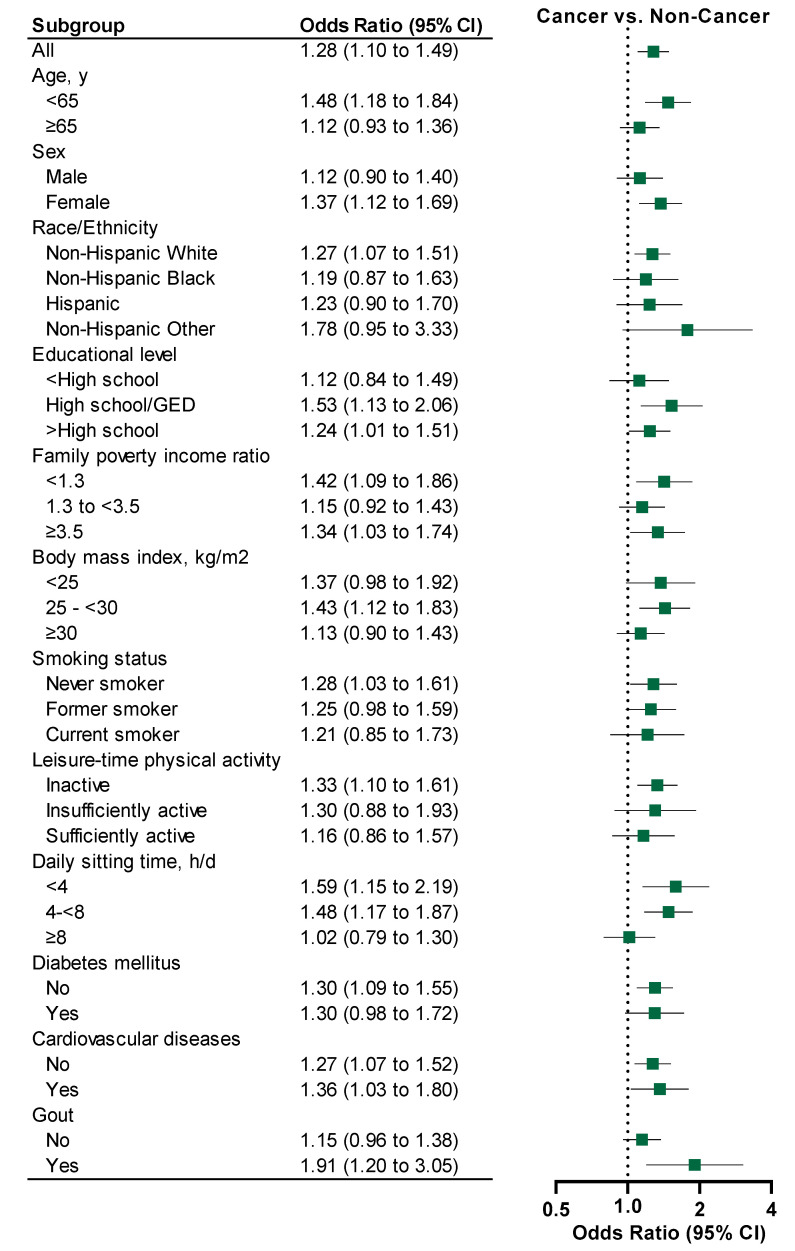
Stratified Odds Ratios and 95% CIs for Kidney Stones Comparing Cancer Survivors With Non-Cancer Adults By Population Characteristics, NHANES 2007–2020 ^a,b^. ^a^ age, sex, race/ethnicity, educational level, family poverty income ratio, body mass index, smoking status, leisure-time physical activity, daily sitting time, history of diabetes, cardiovascular diseases, and gout, pregnancy (female only), hormone therapy (female only), total energy intake and daily intakes of calcium, sodium, water, and alcohol. ^b^ Sample sizes were shown in Table 1.

From 2007–2008 to 2017–2020, the prevalence of kidney stones increased in both non-cancer adults (from 8.5% to 9.2%, *p* for trend = 0.013) and cancer survivors (from 13.1% to 17.3%, *p* for trend = 0.033) (Figure 1). During this period, the prevalence of kidney stones was consistently higher in cancer survivors than in non-cancer adults. The estimated overall prevalence of kidney stones between 2007 and 2020 was 15.8% (95% CI: 14.0–17.5%) in cancer survivors and 9.2% (95% CI: 8.8–9.6%) in non-cancer adults (Table 1). After adjusting for sociodemographic and lifestyle factors and chronic conditions, cancer survivors were more likely to report kidney stones compared to non-cancer adults (OR = 1.28, 95% CI: 1.10–1.49) overall and by sociodemographic and lifestyle factors and health conditions (Figure 2).

### 3.2. Kidney Stones and Sociodemographic, Lifestyle Factors and Chronic Conditions

Among non-cancer adults, older age, male sex, non-Hispanic White, higher BMI, a history of diabetes mellitus, cardiovascular disease, or gout, or ever been pregnant (females only) were associated with a higher likelihood of reporting kidney stones (see Table 2 for multivariable-adjusted OR, 95%; see Table 3 for multivariable adjusted prevalence). Among cancer survivors, male sex, physical inactivity, a history of gout, ever been pregnant (females only), or use of hormone therapy (females only) were associated with a higher likelihood of reporting kidney stones after additional adjustment for age at and years since the first cancer diagnosis. Significant interactions (*p* < 0.05) were observed for age and daily sitting time, indicating their associations with kidney stones differed between non-cancer adults and cancer survivors.

### 3.3. Cancer-Specific Patterns of Kidney Stones

The cancer-specific prevalence of kidney stones was the highest among survivors of kidney cancer (34.7%, 95% CI: 21.0–48.3%), bone and soft tissue cancer (29.9%, 95% CI: 8.0–51.8%), ovarian cancer (29.8%, 95% CI: 15.6–44.0%), and testicular cancer (26.3%, 95% CI: 0–53.6%), followed by bladder cancer (23.0%, 95% CI: 12.8–33.2%), uterine cancer (21.8%, 95% CI: 13.1–30.4%), prostate cancer (20.8%, 95% CI: 16.4–25.2%), and colorectal cancer (19.2%, 95% CI: 13.5–24.8%) (Table 4). The overall prevalence of kidney stones among cancer survivors was significantly higher among male (19.2%, 95% CI: 16.3–22.1%) compared to female survivors (13.5%, 95% CI: 11.3 to 15.6%) (Table 5). Nevertheless, female cancer survivors of upper gastrointestinal tract cancer, lung cancer, and endocrine system cancers (thyroid, lymphoma, and blood and leukemia) reported a higher prevalence of kidney stones than their male counterparts.

**Figure 3:**
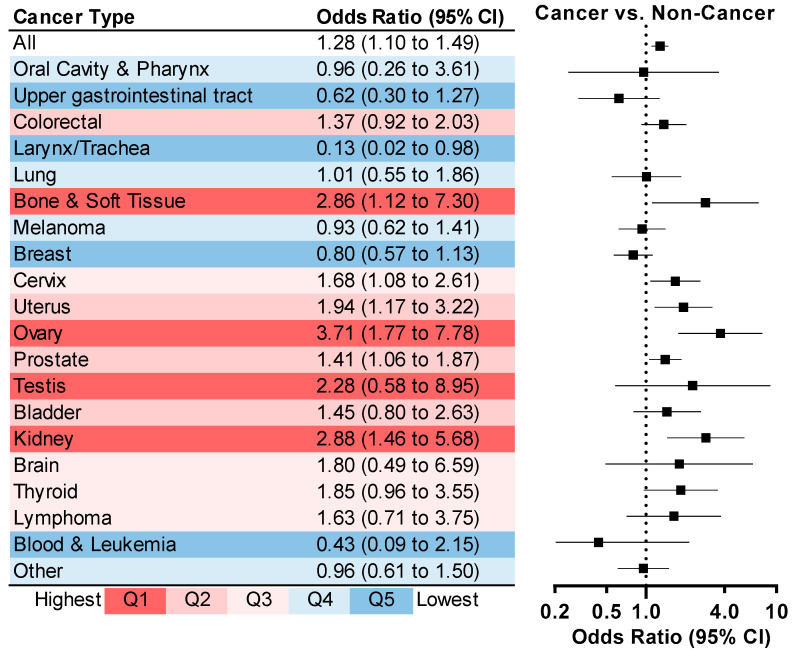
Stratified Odds Ratios and 95% CIs for Kidney Stones Comparing Cancer Survivors With Non-Cancer Adults By Cancer Type, NHANES 2007–2020 ^a^. ^a^ Adjusted for age, sex, race/ethnicity, educational level, family poverty income ratio, body mass index, smoking status, leisure-time physical activity, daily sitting time, history of diabetes, cardiovascular diseases, and gout, pregnancy (female only), hormone therapy (female only), total energy intake and daily intakes of calcium, sodium, water, and alcohol.

In multivariable-adjusted regressions, survivors of ovarian (OR = 3.71, 95% CI: 1.77–7.78), kidney (OR = 2.88, 95% CI: 1.46–5.68), bone and soft tissue (OR = 2.86, 95% CI: 1.12–7.30), uterine (OR = 1.94, 95% CI: 1.17–3.22), and prostate cancers (OR = 1.41, 95% CI: 1.06–1.87) were more likely to report kidney stones compared to non-cancer adults (Figure 3).

## 4. Discussion

In this nationally representative sample of the US population, the estimated prevalence of kidney stones increased in both cancer survivors and non-cancer adults from 2007 to 2020. The prevalence of kidney stones was consistently higher in cancer survivors than in non-cancer adults, with 15.8% of cancer survivors and 9.2% of non-cancer adults reporting kidney stones. A higher prevalence of kidney stones was associated with older age, male sex, non-Hispanic White race/ethnicity, higher BMI, a history of diabetes mellitus, cardiovascular disease, or gout, or having ever been pregnant (females only). The prevalence of kidney stones varied across cancer types and was higher in survivors of kidney, bone, and soft tissue ovarian and testicular cancers. In contrast to the overall sex disparity pattern, female survivors of upper gastrointestinal tract, lung, and endocrine system cancers reported a significantly higher prevalence of kidney stones than their male counterparts.

Epidemiological data on kidney stones in cancer survivors have not been reported in the literature. Our previous study demonstrated that the prevalence of kidney stones steadily increased from 6.5% in the 2007–2008 cycle to 9.4% in the 2017–2018 cycle in a nationally representative sample of general US adults from NHANES [13]. The present analyses reported that the prevalence of kidney stones in non-cancer adults remained high through 2017–2020 and uncovered a higher and increasing burden of kidney stones among cancer survivors. Although the burden of kidney stones has not been well reported, previous studies have highlighted the decline in kidney function and the excess burden of chronic kidney disease (CKD) after a cancer diagnosis [15]. For example, the St. Jude Lifetime Cohort Study, which included 25,530 childhood cancer survivors with a median follow-up of 22.3 years, found that survivors were at increased risk for late-onset kidney failure, particularly among those who received a kidney radiation dose ≥15 Gy, high-dose anthracyclines, any exposure to ifosfamide, or nephrectomy [16]. A retrospective study of 4299 patients undergoing nephrectomy for kidney cancer reported that 28.7% had CKD based on the CKD Epidemiology Collaboration (CKD-EPI) equation [17]. Similarly, Eisenberg et al. conducted a retrospective study of 1631 patients with bladder cancer and found that 46% had CKD prior to surgery [18]. To our knowledge, this study is the first epidemiologic investigation to examine the prevalence, factors, and cancer-specific patterns of kidney stones in cancer survivors. Our findings suggested that cancer survivors were significantly more likely to report having kidney stones than non-cancer adults, even after adjusting for a range of sociodemographic and lifestyle factors and chronic conditions. Of note, our cancer-specific analysis indicated that survivors of certain cancer types experienced a disproportional burden of kidney stones, some contrast with the overall sex disparities. The distinct pattern in kidney stones among cancer survivors cannot be fully explained by surveillance bias due to more aggressive healthcare in this population.

Many cancers have shared risk factors of kidney stones, including advanced age, unhealthy lifestyle, and existing chronic conditions [12]. As such, a higher prevalence of kidney stones was similarly observed among non-cancer adults and cancer survivors of those with older age, a higher BMI, and a history of diabetes mellitus, cardiovascular disease, or gout. These traditional risk factors for kidney stones are more common among cancer survivors who live with a wide range of long-term physical and psychosocial challenges after cancer. Cancer survivors face additional, unique, risk factors due to cancer itself and its treatments [19]. For instance, chemotherapy may increase the risk of kidney stones through hyperuricosuria from elevated uric acid levels, dehydration caused by treatment side effects, and renal tubular damage from certain chemotherapy agents [20,21,22], as supported by our findings of the highest prevalence of kidney stones observed in kidney cancer survivors. The observed higher prevalence of kidney stones in survivors of prostate, bladder, rectal, uterine, cervical, and ovarian cancers could be explained by the exposure to pelvic radiation therapy, which causes direct renal damage, ureteral obstruction from scarring [23], and alterations in urinary pH that promote stones formation [24]. Long-term corticosteroid use in cancer treatment can lead to bone loss and hypercalciuria, increasing the risk of calcium-based kidney stones [25,26], in alignment with a higher prevalence of kidney stones in bone cancer survivors. Cancer surgeries, particularly those involving the gastrointestinal tract, can increase the risk of kidney stones through altered fluid and electrolyte balance, malabsorption of calcium and oxalate leading to elevated urinary oxalate levels, and post-operative dehydration that concentrates urine [27]. The formation of kidney stones from heightened risks associated with cancer treatments is likely to be a late effect, which is often overlooked in clinical practice and research. Further studies are necessary to better understand the mechanisms involved in cancer treatment and the risk of kidney stones. Also, the higher prevalence of kidney stones observed in cancer survivors may be partly explained by greater healthcare utilization, including more frequent contact with clinicians and greater use of diagnostic imaging during cancer diagnosis, treatment, and follow-up [28]. This increased surveillance could lead to higher detection of both symptomatic and asymptomatic stones that might otherwise remain undiagnosed in the general population. In particular, survivors of kidney, ovarian, prostate, and bone/soft tissue cancers often undergo intensive surveillance and repeated imaging (e.g., CT scans, ultrasounds), which increases the likelihood of detecting incidental kidney stones [29].

No specific clinical guidelines are currently available on the prevention and management of kidney stones in cancer survivors. General guidelines from the American Urological Association (AUA) [30] and the European Association of Urology (EAU) [31] emphasize a thorough evaluation, including medical and dietary history, serum chemistries, and urinalysis, to identify risk factors and inform treatment strategies. Our findings suggest that certain cancer survivor groups, such as survivors of kidney, ovarian, prostate, and bone/soft tissue cancers, may warrant particular attention and likely to benefit from an integration of kidney stone surveillance into their survivorship care. Integrating kidney stone risk assessment into survivorship care visits, especially in these high-risk subgroups, could allow earlier identification and intervention [15]. In addition, adapting existing urological guidelines to account for oncology-specific factors (e.g., prior nephrotoxic therapies, surgical history, or radiation exposure) may help mitigate the rising burden of kidney stones in this population. Beyond cancer type, further research is needed to identify kidney stone risk factors that are unique to the cancer survivor population, such as cancer treatment, in addition to traditional risk factors, to refine and adapt existing urological guidelines for oncology settings. Our estimates on prevalence and cancer-specific patterns of kidney stones serve as benchmarks for informing the development of tailored guidelines to establish evidence-based strategies to prevent and manage the growing burden of kidney stones in cancer survivors.

### Limitations

The clear strength of this study is that it analyzed more than 10 years of data from nationally representative samples of US cancer survivors and non-cancer adults, which allowed for population-level estimates and evaluation of temporal trends in patterns of kidney stones. This study also had some limitations. An important limitation of this study is that kidney stone status was self-reported, which may be subject to recall bias and limited accuracy, particularly due to the underreporting of asymptomatic stones, potentially leading to misclassification. Also, we were unable to evaluate the impacts of cancer histology and treatment on kidney stones, as NHANES did not collect data on these clinical variables. Although NHANES is designed to be nationally representative of the non-institutionalized US population, the small sample sizes of certain cancer types may result in unstable estimates that should be interpreted with caution. Also, as this study was based on US NHANES data, the findings may not be generalizable to populations in other countries with different cultural, healthcare, and environmental contexts.

## 5. Conclusions

In the US adult population, the increasing prevalence of kidney stones was consistently higher in cancer survivors than non-cancer adults from 2007 to 2020, affecting more than 15% of cancer survivors. Survivors of certain cancers, including ovarian, kidney, bone and soft tissue, uterine, cervical, and prostate cancers, experienced a higher burden of kidney stones. Future research to elucidate the mechanisms underlying kidney stone formation in cancer survivors is urgently needed to strengthen guidelines for effective clinical management of kidney stones in the oncology setting.

## Figures and Tables

**Table 1:** Prevalence of Kidney Stones Among US Non-Cancer Adults and Cancer Survivors by Sociodemographic and Lifestyle Factors and Chronic Conditions, NHANES 2007–2020 ^a^.

	Non-Cancer Adults			Cancer Survivors		
	Sample Size	Weighted	Prevalence of Kidney Stones, % (95% CI)	Sample Size	Weighted	Prevalence of Kidney Stones, % (95% CI)
	(Weighted%)	Population		**(Weighted%)**	**Population**	
All	40,395 (100)	210,662,296	9.2 (8.8 to 9.6)	3492 (100)	17,607,772	15.8 (14.0 to 17.5)
Age, y						
<65	31,571 (83.7)	176,318,137	8.5 (8.1 to 9.0)	1430 (48.9)	8,607,366	16.4 (13.5 to 19.2)
≥65	8824 (16.3)	34,344,159	12.9 (11.9 to 13.9)	2062 (51.1)	9,000,406	15.2 (13.1 to 17.2)
Sex						
Female	20,696 (51.2)	102,793,435	8.2 (7.7 to 8.8)	1913 (60.3)	6,992,471	13.5 (11.3 to 15.6)
Male	19,699 (48.8)	107,868,861	10.3 (9.7 to 10.9)	1579 (39.7)	10,615,301	19.2 (16.3 to 22.1)
Race/Ethnicity						
Non-Hispanic White	15,228 (64.1)	24,750,299	10.6 (10.0 to 11.2)	1999 (80.5)	1,323,639	16.3 (14.3 to 18.4)
Non-Hispanic Black	9243 (11.7)	135,096,183	5.2 (4.7 to 5.7)	677 (7.5)	14,181,746	9.1 (6.8 to 11.5)
Hispanic	10,287 (15.4)	32,377,212	7.8 (7.2 to 8.3)	538 (6.8)	1,195,831	13.8 (10.5 to 17.1)
Non-Hispanic Other	5637 (8.8)	18,438,602	7.1 (6.1 to 8.0)	278 (5.1)	906,556	19.0 (9.3 to 28.7)
Educational level						
<High school	9637 (15.5)	32,650,750	9.6 (8.8 to 10.4)	773 (13.0)	2,286,309	14.0 (11.0 to 17.1)
High school/GED	9406 (23.8)	50,188,198	9.3 (8.5 to 10.1)	771 (22.1)	3,893,987	16.9 (13.1 to 20.7)
>High school	21,352 (60.7)	127,823,348	9.1 (8.6 to 9.7)	1948 (64.9)	11,427,476	15.7 (13.4 to 18.0)
Family poverty income ratio						
<1.3	11,521 (20.1)	42,372,259	8.6 (7.9 to 9.2)	848 (15.5)	2,733,864	16.0 (12.8 to 19.2)
1.3 to <3.5	18,097 (41.0)	86,396,421	9.6 (9.0 to 10.2)	1636 (42.6)	7,504,040	15.3 (12.7 to 17.8)
≥3.5	10,777 (38.9)	81,893,616	9.2 (8.5 to 9.9)	1008 (41.9)	7,369,868	16.1 (13.1 to 19.2)
Body mass index, kg/m^2^						
<25	10,994 (28.3)	59,567,866	6.4 (5.7 to 7.0)	851 (24.3)	4,270,207	12.0 (8.9 to 15.1)
25–<30	14,377 (35.3)	74,354,693	8.8 (8.2 to 9.5)	1345 (38.1)	6,705,309	15.8 (13.0 to 18.6)
≥30	15,024 (36.4)	76,739,737	11.9 (11.1 to 12.6)	1296 (37.7)	6,632,256	18.1 (15.1 to 21.1)
Smoking status						
Never smoker	23,178 (56.9)	119,891,617	8.4 (7.9 to 9.0)	1632 (47.6)	8,377,508	15.3 (12.8 to 17.8)
Former smoker	9104 (23.5)	49,490,164	11.1 (10.2 to 12.0)	1298 (36.3)	6,399,613	16.9 (14.0 to 19.8)
Current smoker	8113 (19.6)	41,280,515	9.3 (8.5 to 10.2)	562 (16.1)	2,830,651	14.5 (10.3 to 18.7)
Leisure-time physical activity						
Inactive	21,352 (46.2)	97,304,212	10.4 (9.8 to 11.0)	2145 (54.9)	9,660,756	17.0 (14.6 to 19.3)
Insufficiently active	5801 (16.0)	33,615,289	8.7 (7.7 to 9.8)	445 (13.8)	2,427,753	15.8 (11.2 to 20.4)
Sufficiently active	13,242 (37.9)	79,742,795	8.1 (7.4 to 8.7)	902 (31.3)	5,519,263	13.6 (10.5 to 16.7)
Daily sitting time, h/d						
<4	11,862 (25.1)	52,795,175	8.8 (8.1 to 9.6)	741 (18.7)	3,298,830	17.1 (13.0 to 21.1)
4–<8	15,882 (39.8)	83,854,203	9.0 (8.4 to 9.7)	1515 (41.9)	7,384,669	17.0 (14.1 to 19.9)
≥8	12,651 (35.1)	74,012,918	9.8 (9.0 to 10.5)	1236 (39.3)	6,924,273	13.8 (11.3 to 16.3)
Diabetes mellitus						
No	35,164 (90.6)	190,925,231	8.4 (8.0 to 8.8)	2701 (81.7)	14,389,302	14.4 (12.5 to 16.3)
Yes	5231 (9.4)	19,737,065	17.1 (15.5 to 18.7)	791 (18.3)	3,218,470	21.9 (17.7 to 26.1)
Cardiovascular diseases						
No	36,227 (92.1)	194,017,838	8.6 (8.2 to 9.0)	2619 (79.1)	13,924,477	14.5 (12.6 to 16.4)
Yes	4168 (7.9)	16,644,458	16.5 (14.9 to 18.1)	873 (20.9)	3,683,295	20.4 (16.4 to 24.3)
Gout						
No	30,598 (82.0)	172,784,482	9.0 (8.5 to 9.4)	2393 (75.0)	13,212,348	13.9 (12.0 to 15.8)
Yes	1385 (3.1)	6,590,860	16.9 (14.1 to 19.7)	268 (7.3)	1,276,682	30.6 (22.0 to 39.3)

^a^ Estimates were weighted to be nationally representative.

**Table 2:** Weighted Logistic Regression Models of Kidney Stones Among US Non-Cancer Adults and Cancer Survivors, NHANES 2007–2020 ^a^.

	Odds Ratio (95% CI)		*p* for Interaction ^c^
	Non-Cancer Adults (N = 40,395)	Cancer Survivors ^b^ (N = 3492)	
Age, y	1.02 (1.01 to 1.02)	0.99 (0.98 to 1.01)	<0.001
Sex			
Female	1 [Reference]	1 [Reference]	0.450
Male	1.51 (1.28 to 1.78)	3.48 (2.08 to 5.81)	
Race/Ethnicity			
Non-Hispanic White	1 [Reference]	1 [Reference]	0.067
Non-Hispanic Black	0.45 (0.39 to 0.50)	0.43 (0.30 to 0.62)	
Hispanic	0.76 (0.67 to 0.85)	0.75 (0.52 to 1.08)	
Non-Hispanic Other	0.73 (0.62 to 0.86)	1.10 (0.63 to 1.93)	
Educational level			
<High school	1 [Reference]	1 [Reference]	0.766
High school/GED	0.96 (0.83 to 1.11)	1.19 (0.82 to 1.74)	
>High school	1.05 (0.92 to 1.20)	1.18 (0.84 to 1.67)	
Family poverty income ratio			
<1.3	1 [Reference]	1 [Reference]	0.770
1.3 to <3.5	1.01 (0.90 to 1.13)	0.83 (0.59 to 1.16)	
≥3.5	0.93 (0.82 to 1.07)	0.95 (0.64 to 1.42)	
Body mass index, kg/m^2^			
<25	1 [Reference]	1 [Reference]	0.279
25–<30	1.30 (1.13 to 1.49)	1.30 (0.90 to 1.89)	
≥30	1.76 (1.54 to 2.01)	1.31 (0.90 to 1.92)	
Smoking status			
Never smoker	1 [Reference]	1 [Reference]	0.741
Former smoker	1.00 (0.88 to 1.12)	0.97 (0.72 to 1.30)	
Current smoker	1.14 (1.00 to 1.30)	0.79 (0.51 to 1.24)	
Leisure-time physical activity			
Inactive	1 [Reference]	1 [Reference]	0.899
Insufficiently active	0.91 (0.78 to 1.05)	0.85 (0.56 to 1.30)	
Sufficiently active	0.92 (0.82 to 1.04)	0.66 (0.48 to 0.93)	
Daily sitting time, h/d			
<4	1 [Reference]	1 [Reference]	0.018
4–<8	0.91 (0.80 to 1.03)	0.97 (0.67 to 1.41)	
≥8	0.97 (0.85 to 1.10)	0.69 (0.47 to 1.02)	
Diabetes mellitus			
No	1 [Reference]	1 [Reference]	0.298
Yes	1.55 (1.35 to 1.78)	1.44 (1.06 to 1.96)	
Cardiovascular diseases			
No	1 [Reference]	1 [Reference]	0.550
Yes	1.26 (1.09 to 1.46)	1.29 (0.95 to 1.76)	
Gout			
No	1 [Reference]	1 [Reference]	0.237
Yes	1.29 (1.05 to 1.60)	2.28 (1.49 to 3.49)	
Pregnancy (female only)			
Never	1 [Reference]	1 [Reference]	0.929
Ever	1.23 (1.03 to 1.46)	2.12 (1.29 to 3.50)	
Hormone therapy (female only)			
Never	1 [Reference]	1 [Reference]	0.547
Ever	1.08 (0.90 to 1.30)	1.57 (1.06 to 2.31)	

^a^ Estimates were weighted to be nationally representative. The models were age, sex, race/ethnicity, educational level, family poverty income ratio, body mass index, smoking status, leisure-time physical activity, daily sitting time, history of diabetes, cardiovascular diseases, and gout, pregnancy (female only), hormone therapy (female only), total energy intake and daily intakes of calcium, sodium, water, and alcohol. ^b^ The models were additionally adjusted for age at and years since the first cancer diagnosis. ^c^ The significant interaction indicated that the effect of correlate is different between US non-cancer adults and cancer survivors.

**Table 3:** Multivariable-Adjusted Prevalence of Kidney Stones Among US Non-Cancer Adults and Cancer Survivors, NHANES 2007–2020 ^a^.

	Non-Cancer Adults (N = 40,395)	Cancer Survivors (N = 3492)
	Prevalence of Kidney Stone	Prevalence of Kidney Stone
	Weighted% (95% CI)	Weighted% (95% CI)
All	9.4 (9.0 to 9.9)	16.7 (14.8 to 18.5)
Age, y		
<65	9.4 (8.9 to 9.9)	16.5 (14.4 to 18.6)
≥65	9.7 (8.5 to 10.9)	16.8 (14.9 to 18.7)
Sex		
Male	12 (11.1 to 12.8)	19.6 (17.5 to 21.7)
Female	7.1 (6.4 to 7.7)	14.7 (12.8 to 16.6)
Race/Ethnicity		
Non-Hispanic White	10.9 (10.3 to 11.5)	17.5 (15.6 to 19.4)
Non-Hispanic Black	4.4 (3.8 to 5.0)	11.0 (9.1 to 13.0)
Hispanic	7.8 (7.0 to 8.5)	14.4 (12.3 to 16.4)
Non-Hispanic Other	8 (6.8 to 9.3)	14.6 (12.3 to 17.0)
Educational level		
<High school	9.2 (8.1 to 10.2)	16.3 (14.3 to 18.4)
High school/GED	8.6 (7.7 to 9.5)	15.8 (13.7 to 17.8)
>High school	9.8 (9.3 to 10.4)	17.0 (15.1 to 18.9)
Family poverty income ratio		
<1.3	8.9 (8.2 to 9.7)	16.1 (14.1 to 18.1)
1.3 to <3.5	9.6 (9.0 to 10.3)	16.8 (14.9 to 18.8)
≥3.5	9.5 (8.7 to 10.3)	16.7 (14.7 to 18.7)
Body mass index, kg/m^2^		
<25	7.1 (6.4 to 7.8)	14.2 (12.2 to 16.2)
25–< 30	9.3 (8.5 to 10)	16.4 (14.4 to 18.3)
≥30	11.4 (10.6 to 12.2)	18.5 (16.5 to 20.4)
Smoking status		
Never smoker	9.1 (8.5 to 9.6)	16.2 (14.3 to 18.2)
Former smoker	9.9 (9.0 to 10.8)	17.0 (15.0 to 19.0)
Current smoker	10.0 (9.0 to 11.0)	17.1 (15.0 to 19.2)
Leisure-time physical activity		
Inactive	10.2 (9.5 to 10.9)	17.3 (15.4 to 19.2)
Insufficiently active	8.5 (7.5 to 9.6)	15.6 (13.5 to 17.7)
Sufficiently active	8.9 (8.2 to 9.6)	16.0 (14.1 to 18.0)
Daily sitting time, h/d		
<4	9.9 (9.1 to 10.7)	17.2 (15.1 to 19.2)
4–< 8	9.2 (8.5 to 9.9)	16.5 (14.5 to 18.4)
≥8	9.4 (8.7 to 10.1)	16.6 (14.7 to 18.6)
Diabetes mellitus		
No	8.9 (8.4 to 9.3)	15.6 (13.7 to 17.5)
Yes	15.0 (13.3 to 16.6)	21.7 (19.3 to 24.0)
Cardiovascular diseases		
No	9.1 (8.7 to 9.6)	15.8 (13.9 to 17.7)
Yes	13.1 (11.4 to 14.8)	19.8 (17.4 to 22.2)
Gout		
No	9.2 (8.7 to 9.7)	16.2 (14.3 to 18.0)
Yes	15.3 (12.4 to 18.2)	22.2 (18.8 to 25.7)

^a^ Adjusted for age, sex, race/ethnicity, educational level, family poverty income ratio, body mass index, smoking status, leisure-time physical activity, daily sitting time, history of diabetes, cardiovascular diseases, and gout, pregnancy (female only), hormone therapy (female only), total energy intake and daily intakes of calcium, sodium, water, and alcohol.

**Table 4:** Cancer-Specific Prevalence of Kidney Stones Among US Adult Cancer Survivors, NHANES 2007–2020 ^a^.

Cancer Type	N	Prevalence of Kidney Stone
	Weighted population	Weighted% (95% CI)
All	3492	15.8
	17,607,772	(14.0 to 17.5)
Oral Cavity and Pharynx	26	14.5
	149,406	(0 to 30.5)
Upper gastrointestinal tract	117	8.0
	456,521	(2.7 to 13.2)
Colorectal	351	19.2
	1,429,235	(13.5 to 24.8)
Larynx/Trachea	22	1.7
	103,778	(0 to 5.3)
Lung	127	13.8
	523,098	(6.9 to 20.8)
Bone and Soft Tissue	37	29.9
	171,209	(8.0 to 51.8)
Melanoma	304	13.7
	2,172,236	(8.8 to 18.5)
Breast	693	10.2
	3,909,645	(7.3 to 13.2)
Cervix	258	16.3
	1,639,971	(10.2 to 22.3)
Uterus	195	21.8
	983,724	(13.1 to 30.4)
Ovary	115	29.8
	632,493	(15.6 to 44.0)
Prostate	710	20.8
	2,512,044	(16.4 to 25.2)
Testis	23	26.3
	282,685	(0 to 53.6)
Bladder	121	23.0
	483,796	(12.8 to 33.2)
Kidney	98	34.7
	376,685	(21.0 to 48.3)
Brain	24	16.1
	106,609	(0 to 35.1)
Thyroid	115	18.2
	660,376	(8.0 to 28.4)
Lymphoma	107	16.3
	497,990	(5.1 to 27.5)
Blood and Leukemia	73	5.6
	383,075	(0 to 14.2)
Other	272	12.9
	1,659,134	(7.7 to 18.1)

^a^ Estimates were weighted to be nationally representative. The color scale indicated the quintile of prevalence of kidney stones across different types of cancer.

**Table 5:** Cancer-Specific Prevalence of Kidney Stones Among US Adult Cancer Survivors By Sex, NHANES 2007–2020 ^a^.

Cancer Type	Prevalence, Weighted% (95% CI)	
	Male	Female
All	19.2	13.5
	(16.3 to 22.1)	(11.3 to 15.6)
Oral Cavity and Pharynx	29.3	
	(0.1 to 58.4)	
Upper gastrointestinal tract	4.5	11.5
	(1.0 to 8.1)	(1.4 to 21.5)
Colorectal	25.6	14.4
	(15.9 to 35.3)	(7.8 to 21)
Larynx/Trachea	1.8	
	(0 to 5.9)	
Lung	7.9	18.9
	(1.1 to 14.6)	(7.3 to 30.4)
Bone and Soft Tissue	34.3	20.3
	(5.1 to 63.4)	(0 to 50.3)
Melanoma	20.0	5.6
	(12.1 to 28.0)	(2 to 9.2)
Breast		10.3
		(7.3 to 13.2)
Cervix		16.3
		(10.2 to 22.3)
Uterus		21.8
		(13.1 to 30.4)
Ovary		29.8
		(15.6 to 44.0)
Prostate	20.8	
	(16.4 to 25.2)	
Testis	26.3	
	(0 to 53.6)	
Bladder	24.3	19.9
	(11.6 to 36.9)	(3.1 to 36.8)
Kidney	36.5	30.2
	(19.4 to 53.6)	(7.9 to 52.4)
Brain	17.8	14.7
	(0 to 56.1)	(0 to 37.1)
Thyroid	1.9	22.2
	(0 to 6)	(9.8 to 34.5)
Lymphoma	13.3	18.9
	(0 to 26.7)	(1.4 to 36.5)
Blood and Leukemia	0.7	16.1
	(0 to 2)	(0 to 41.1)
Other	15.8	9.8
	(7.1 to 24.5)	(4.3 to 15.3)

^a^ Estimates were weighted to be nationally representative.

## Data Availability

The datasets generated during and/or analyzed during the current study are available in the National Center for Health Statistics repository. https://www.cdc.gov/nchs/nhanes/index.html (accessed on 3 December 2022).
